# Intranasal insulin reverts central pathology and cognitive impairment in diabetic mother offspring

**DOI:** 10.1186/s13024-017-0198-4

**Published:** 2017-08-02

**Authors:** Juan Jose Ramos-Rodriguez, Daniel Sanchez-Sotano, Alberto Doblas-Marquez, Carmen Infante-Garcia, Simon Lubian-Lopez, Monica Garcia-Alloza

**Affiliations:** 1 Division of Physiology, School of Medicine Universidad de Cadiz, Plaza Fragela sn, 4 piso 410, Cadiz, Spain; 2 Instituto de Investigación Biomédica e Innovación en Ciencias Biomédicas de la Provincia de Cádiz (INiBICA), Cadiz, Spain; 3 Division of Neonatology, Hospital Universitario Puerta del Mar, Cadiz, Spain; 4 Division of Pediatrics, Hospital Universitario Puerta del Mar, Cadiz, Spain

**Keywords:** Diabetes, Offspring, Insulin, Tau, Haemorrhage, Inflammation

## Abstract

**Background:**

Adverse effects in diabetic mothers offspring (DMO) are a major concern of increasing incidence. Among these, chronic central complications in DMO remain poorly understood, and in extreme cases, diabetes can essentially function as a gestational brain insult. Nevertheless, therapeutic alternatives for DMO are limited.

**Methods:**

Therefore, we have analyzed the central long-term complications in the offspring from CD1 diabetic mothers treated with streptozotozin, as well as the possible reversion of these alterations by insulin administration to neonates. Brain atrophy, neuronal morphology, tau phosphorylation, proliferation and neurogenesis were assessed in the short term (P7) and in the early adulthood (10 weeks) and cognitive function was also analyzed in the long-term.

**Results:**

Central complications in DMO were still detected in the adulthood, including cortical and hippocampal thinning due to synaptic loss and neuronal simplification, increased tau hyperphosphorylation, and diminished cell proliferation and neurogenesis. Additionally, maternal diabetes increased the long-term susceptibility to spontaneous central bleeding, inflammation and cognition impairment in the offspring. On the other hand, intracerebroventricular insulin administration to neonates significantly reduced observed alterations. Moreover, non-invasive intranasal insulin reversed central atrophy and tau hyperphosphorylation, and rescued central proliferation and neurogenesis. Vascular damage, inflammation and cognitive alterations were also comparable to their counterparts born to nondiabetic mice, supporting the utility of this pathway to access the central nervous system.

**Conclusions:**

Our data underlie the long-term effects of central complications in DMO. Moreover, observed improvement after insulin treatment opens the door to therapeutic alternatives for children who are exposed to poorly controlled gestational diabetes, and who may benefit from more individualized treatments.

## Background

Gestational diabetes affects 3–10% of pregnant women [1] and epidemiological and animal studies have previously shown that the risk of adverse maternal and perinatal outcomes continuously increases with maternal glycaemia [2]. Following this idea, malformation and mortality rates are reportedly two to five fold higher in diabetic mothers offspring (DMO) (for review [3]). Moreover, these foetal and neonatal complications seem to continue at later stages and have been largely studied at metabolic level [4]. Central nervous system (CNS) complications in DMO are also receiving attention in recent years and many studies have focused on hypothalamic alterations in relation with future metabolic disorders [5, 6]. Prolonged hyperglycaemia during critical periods of development underlie malformations in the foetal brain [7] and insulin is also an important regulator of developmental and cognitive functions in the CNS [8]. Optimal control of glucose levels during pregnancy is highly pursued, however, short and long-term related complications are still present and in extreme cases, diabetes has been suggested to essentially function as a gestational brain insult [9] resulting in behavioural problems, language impairment or cognitive development deficits [9, 10]. However, to our knowledge exhaustive chronic evaluation of the CNS has not been performed, and the study of therapeutic options should also be addressed. Therefore, we have assessed short and long-term metabolic and central complications in DMO. Moreover, we have attempted to reverse observed alterations by treating DMO with insulin. Although previous studies have treated diabetic mothers with insulin to counterbalance disturbances in the offspring, to our knowledge no preceding studies have directly administered intracerebroventricular (ICV) or intranasal (IN) insulin to DMO. In our hands, DMO presented metabolic alterations in the adulthood, including modified glucose and insulin levels in glucose tolerance tests, while insulin administration significantly improved this aspect. When we analyzed the CNS, DMO presented long-term cortical and hippocampal thinning due to neuronal simplification and synaptic loss, accompanied by reduced central proliferation and neurogenesis, while insulin treatments reverted these effects. Also, spontaneous central bleeding in DMO was significantly improved after insulin administration, and markers of neuronal damage, such as tau hyperphosphorylation were controlled by insulin. Cognition impairment was significantly reversed after insulin treatment. An overall improvement was observed after both, IN and ICV insulin administration, nevertheless, IN pathway lead to a more robust recovery, supporting the utility of this approach to guarantee insulin access to the CNS. Altogether our data could help to elucidate the underlying central complications in DMO and open the door to therapeutic alternatives for children who are exposed to poorly controlled gestational diabetes.

## Methods

### Animals and treatments

Two months old CD1 breeders were treated with streptozotozin (STZ) (50 mg/Kg) for 5 consecutive days [11] prior crossing with healthy males. Both female and male offspring from diabetic and control mothers were randomly divided and sacrified immediately after birth or at P7. A set of animals received unilateral ICV insulin (ICV-Ins) injection at P7 [12, 13]. Briefly, mice were anesthetized with isoflurane (Astrazeneca, Spain) and placed in a sterotaxic device (David-Kopf Instruments, Tujunga, CA, USA). ICV administration consisted of 1 μl of insulin (5 mIU) in PBS, at the following coordinates: AP -3 mm, ML -1 mm y DV +4 mm from Bregma. Injections were performed with a 5 μl Hamilton syringe (Hamilton Company, Bonaduz, Switzerland) at a constant flow rate of 0.2 μl/min for 5 min. A delay of 5 min was allowed before complete retraction of the needle to minimize aspiration of the toxin. Sham operated mice followed the same procedure but only PBS was injected. A second group received IN insulin (IN-Ins), for 7 consecutive days (P7-P13) as previously described [14, 15]. Briefly, mice received 1 IU/day of insulin in PBS. Mice were lightly anesthetized with isoflurane and held gently on their backs. A 10 μl pipette was used for intranasal administration (1.25 μl of insulin every minute) alternating nostrils. Insulin drops were placed in the opening of the nostril, allowing the mouse to snort each drop into the nasal cavity. A total of 5 μl of insulin were delivered over a course of 4 min. Control mice received PBS following a similar approach. Long-term effects of the ICV-Ins and IN-Ins treatments was assessed at 10 weeks of age. For proliferation and neurogenesis studies animals received BrdU ip (70 mg/Kg) for 3 consecutive days immediately before sacrifice.

**Fig. 1:**
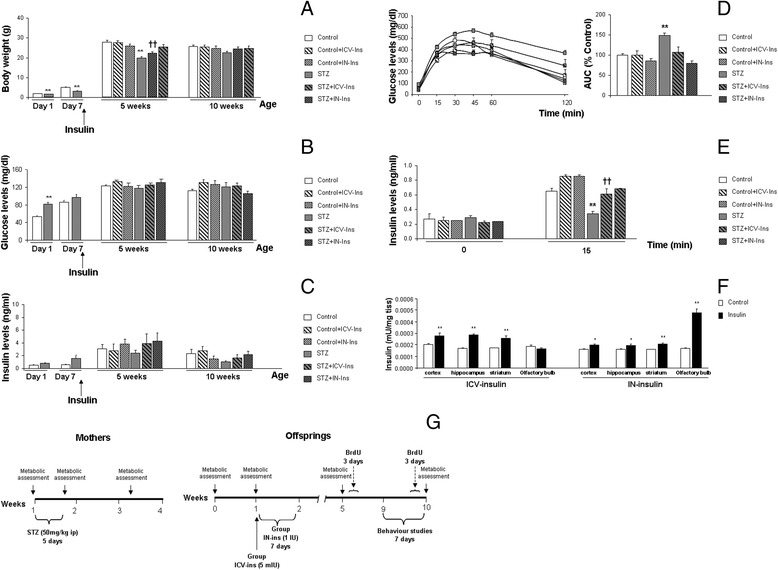
Short and long-term metabolic characterization of DMO after insulin treatments. **a** Body weight was reduced in DMO at P1 (**p < 0.01 vs. Control), P7 (**p < 0.01 vs. Control) and 5 weeks (**p < 0.01 vs. rest of the groups) while differnces were no longer observed and 10 weeks of age (*p* = 0.146). **b** Glucose levels (mg/dl) were reduced at P1 (**p < 0.01 vs. Control) and no further differences were detectable P7 (*p* = 0.171), 5 weeks (*p* = 0.424) and 10 weeks (*p* = 0.158). **c** Insulin levels (ng/ml) were slighly reduced at P1 and P7 although differences did not reach statiscital sifnificance (*p* = 0.064 and *p* = 0.077). No differences were observed in insulin levels at 5 (*p* = 0.716) or 10 weeks (*p* = 0.451) (P1 Control *n* = 6 and SZT *n* = 4; P7 Control *n* = 7 and STZ *n* = 9; 5 weeks Control *n* = 15, Control + ICV-Ins *n* = 8, Control + IN-Ins *n* = 8, STZ *n* = 8, STZ + ICV-Ins *n* = 6 and STZ + IN-Ins *n* = 7; 10 weeks Control *n* = 27, Control + ICV-Ins *n* = 17, Control + IN-Ins *n* = 8, STZ *n* = 18, STZ + ICV-Ins *n* = 10 and STZ + IN-Ins *n* = 6). **d** GTT measured as AUC was signifcantly reduced in DMO (% Control) (**p < 0.01 vs. rest of the groups) (Control *n* = 13, Control + ICV-Ins *n* = 8, Control + IN-Ins *n* = 6, STZ *n* = 13, STZ + ICV-Ins *n* = 8 and STZ + IN-Ins *n* = 5). **e** Plasma insulin levels during GTT were not affected at time 0 (*p* = 0.866), while insulin treatment significantly increased insulin levels at time 15 (**p < 0.01 vs. rest of the groups, ††*p* < 0.01 vs. Control + IN-Ins) (Control *n* = 13, Control + ICV-Ins *n* = 7, Control + IN-Ins *n* = 6, STZ *n* = 13, STZ + ICV-Ins *n* = 8 and STZ + IN-Ins *n* = 5). Student t-test for independent samples was used when 2 populations were under study (P1 and P7) and one-way ANOVA, followed by Tuckey b or Tamhane test, was used when 6 groups were under study (5 and 10 weeks). **f** Central insulin levels were increased in the brain after IN and ICV administration. **g** Experimental timeline including all groups under study

DMO were assigned to foster mothers immediately after birth to guarantee regular postnatal feeding and development. Groups and treatments are presented in Fig. 1g. Metabolic and postmortem characterization included 4–6 animals per group at P1 and 4–7 mice per group at P7. Five weeks old assessment included 5–15 mice and 10 weeks old studies included 6–28 mice per group for behavioural and metabolic assessment and 3–10 mice per group in postmortem studies. Individual sample size for each experiment is detailed in figure legends. In order to guarantee the access of insulin to the brain, a set of animals (*n* = 3–4) was treated with ICV-Ins or IN-Ins, as described above, and scarified to measure brain insulin levels in the olfactory bulb, cortex, hippocampus and striatum. All experimental procedures were approved by Junta de Andalucia, (Guidelines for Care and Use of Experimental Animals, European Commission Directive 2010/63/UE and Spanish Royal Decree 53/2013).

### Brain insulin levels

Brain insulin levels were measured in the olfactory bulb, cortex, hippocampus and striatum of a set of mice, after ICV-Ins and IN-Ins, to guarantee the access to the brain after both administration pathways. Briefly, animals received an overdose of pentobarbital (60 mg/Kg) (Sigma, St. Louis, MO, USA Sigma MO). Selected brain regions were harvested and snap frozen until used. Tissue was homogenised in lysis buffer (CellSignaling, USA) supplemented with a protease inhibitor cocktail (Sigma, USA) and insulin levels were determined by human enzyme-linked immunosorbent assay (Mercodia, Spain Iso-Insulin ELISA 10–1128-01).

### Metabolic characterization

Body weight was determined in the mothers before STZ administration and on the day of birth, and weight gain was calculated. Postprandial glucose levels were measured before STZ treatment, one week after STZ administration, after crossing and the day of birth. Insulin levels were also measured immediately before STZ administration and the day of birth. Body weight, glucose levels and insulin levels were determined in all offspring (both male and female) the day of birth, and on P7. Endpoint experiments were programmed to determine glucose and insulin levels due to the volume of blood required. Briefly, animals were deeply anesthetized with pentobarbital (60 mg/kg) and blood was withdrawn by cardiac puncture. Glucose an insulin levels were also determined after ICV-Ins or IN-Ins administration, at 5 and 10 weeks of age. Additionally, at 10 weeks of age, before sacrifice, intraperitoneal glucose tolerance test (GTT) (2 g/Kg body weight, Sigma, OR; USA) was performed [16]. Blood glucose levels were measured in mg/dl using the glucometer Optium Xceed (Abbott, United Kingdom). Plasma insulin levels were measured in ng/ml using ultrasensitive mouse enzyme-linked immunosorbent assay (Mercodia, Spain Mouse Insulin ELISA 10–1247-01).

### Motor activity and new object discrimination (NOD) task

Seven days prior to sacrifice we assessed spontaneous locomotor activity in male and female offspring, measuring the distance travelled by mice for 30 min on day 1, before the commencement of the NOD test. Integrated episodic memory for “what”, “where” and “when” paradigms were analyzed as previously described [17].

### Morris water maze (MWM)

Spatial cognition assessment commenced in the same mice the day after concluding the NOD test. Acquisition consisted in 4 trials/day (60 s/trial) for 4 days, with the platform submerged in quadrant 2. If the animal did not find the platform it was placed on it for 10 s. Retention started a day after and consisted in a single trial with the platform removed [17]. Time required to locate the platform in the acquisition phase, percentage of time spent in quadrant 2 during the retention phase and swim speed were analyzed using Smart software, (Panlab, Spain).

### Tissue processing

Cortex and hippocampus from the right hemisphere, from both male and female offspring, were dissected and snap frozen for cresyl violet, Prussian blue, caspase activity and western blot studies. Left hemispheres, from both male and female offspring, were fixed in paraformaldehyde for 2 weeks before 30 μm coronal sections were obtained. In case of ICV treated mice, only ipsilateral hemispheres were used and brains from males and females were either dissected or snap frozen. Similarly, P1 and P7 tissue was snap frozen for biochemical determinations.

### Cresyl violet and Prussian blue staining

Brain morphology was analyzed after cresyl violet staining in sections (1.5 to −3.5 mm from Bregma). Cortex and hippocampus thickness were measured using Adobe Photoshop and Image J software as previously described [18].

Presence of haemorrhages was conducted using Prussian blue iron staining and neutral red counterstain in consecutive sections. Sections were imaged with an Olympus Bx60 microscope (Japan) and an Olympus DP71 camera. Images were analyzed using Adobe PhotoShop and Image J softwares to quantify number of haemorrhages, individual haemorrhage size, and haemorrhage burden in the cortex and hippocampus [18].

### Caspase activation

Caspases 3/7 activity was analyzed in cortical homogenates from all groups in this study using the Caspase-Glo 3/7 assay (Promega, Madrid, Spain), following manufacturer’s indications, as previously described [19].

### Inmunohistochemistry studies: 5-bromo-3-deoxyuridin, doublecortin, NeuN and microglia

5-bromo-3-deoxyuridine (BrdU) and doublecortin (DCX) immunohistochemistry was performed in the cortex, hippocampus and the subventricular zone (SVZ) (0.5, and 0.0 mm from Bregma). Anti-BrdU 1:100 (Dako, Barcelona, Spain) and anti-DCX 1:400 (SantaCruz Biotechnology, Santa Cruz, CA, USA) were used as primary antibodies and Alexa Fluor 594 and Alexa Fluor 488 (1:100) (Invitrogen, Carlsbad, CA, USA) as secondary antibodies [18]. DCX burden (percentage of area covered by DCX-positive cells) was quantified in the SVZ [18] and number of individual DCX- and BrdU-positive cells were quantified in the cortex and hippocampus using Image J software.

NeuN and microglia immunohistochemistry was performed as described [20]. Anti-Iba1 (Wako, Osaka,Japan) (1:2000) or anti-NeuN (Chemicon, CA, USA) (1:200) (Invitrogen, Carlsbad, CA, USA) were used as primary antibodies and Alexa Fluor 488 and Alexa Fluor 594 (1:200) as secondary antibodies. DAPI 1 mg/ml (Sigma) (1:2000) counterstain was used. The percentage of NeuN-positive cells (normalized by total cells stained with DAPI) was quantified as previously described [21]. Number of microglia cells, individual microglia size and burden were quantified in the cortex and hippocampus using Image software [20].

### Golgi-Cox staining

Neuronal complexity was measured by Golgi-Cox staining, using Rapid Golgi Stain Kit (FD Neurotechnologies, USA. Ref: PK401). Kit instructions were followed as previously described [20] in all of our animals (both male and female). Neuronal complexity was analyzed by sholl analysis in 10 μm concentric circles from neuronal soma. Spine density was calculated (spines/10 μm) [20]. Ratios of curvature were calculated by dividing the end-to-end distance of a dendrite segment by the total length between the two segment ends. Analysis was completed using Image J software, as previously described [22].

### Western blot for tau, Akt and synaptophysin

Tau, tau phosphorylation, Akt and phospho-Akt were measured in cortical and hippocampal samples as previously described [23] at P1, P7 and 10 weeks. To prove insulin central effects early Phospho-Akt/total Akt ratios are were also measured in a set of offspring 4 h after ICV or last intranasal insulin administration. Phospho-tau clone AT8 (1:1000, Fisher Scientific, MA, USA), phospho-Akt (Ser473) (1:1000) (Cell signaling, USA), anti-synaptophysin (1:1000) (Zymed, USA), anti-total Akt antibody (1:1000) (cell signaling, USA) and anti-total tau antibody (1:1000) (DAKO, Denmark) were used. Optical density was semi-quantified after normalizing to β-actin (1:2,500,000) (Sigma, USA), using Image J software. Phospho-tau/total tau, phospho-Akt/total Akt ratios and synaptophysin levels were represented as percentage of Control values.

### Statistical analysis

Student t test for independent samples or one-way ANOVA, followed by Tuckey b test or Tamhane tests as required, were used. Two-way ANOVA (groupXday) was used to analyze the the MWM test. SPSS v.15 software was used for all statistical analysis.

## Results

### Metabolic characterization

No differences were detected among mothers before STZ treatment when body weight (*p* = 0.850), glucose levels (*p* = 0.798) or insulin levels were compared (*p* = 0.382) (Table 1). We detected a significant increase of glycaemia one week after STZ treatment (**p = 0.005 vs. Control). At the date of birth body weight was significantly lower in STZ-treated mothers (**p = 0.003 vs. Control) and the increase of body weight was also significantly compromised (**p = 0.003 vs. Control). The day of birth insulin levels were lower in STZ-treated mothers (**p = 0.005 vs. Control) and glucose levels were highly increased (**p < 0.001 vs. Control) (Table 1).

**Table 1:** Metabolic assessment of STZ-treated mothers

	Treatment	
	Control	STZ
Initial body weight (g)	30.72 ± 1.06	31.11 ± 1.45
Body weight (g) at birth	42.31 ± 1.31	35.76 ± 1.09**
Increase in body weight (g)	11.6 ± 1.45	5.43 ± 0.85**
Initial insulin levels (ng/ml)	0.63 ± 0.17	0.94 ± 0.24
Insulin levels (ng/dl) at birth	0.44 ± 0.14	0.03 ± 0.01
Initial glucose levels (mg/dl)	111.20 ± 5.08	112.75 ± 3.46
Glucose levels (mg/dl) before crossing	100.60 ± 10.54	184.38 ± 17.67**
Glucose levels (mg/dl) after crossing	111.40 ± 8.90	487.27 ± 44.27**
Glucose levels (mg/dl) at birth	94.00 ± 3.05	500.38 ± 39.29**

Initial body weight (*p* = 0.850), glucose levels (*p* = 0.798) or insulin levels (*p* = 0.382) were similar in both groups before STZ administration. Glucose levels immediately before crossing (one week after commencement of the treatment) were increased in STZ treated mice (**p = 0.005 vs. Control). The date of birth, body weight (**p = 0.003 vs. Control) and the increase of body weight (**p = 0.003 vs. Control) were significantly lower in STZ-treated mothers. The day of birth insulin levels were lower in STZ-treated animals (**p = 0.005 vs. Control) while glucose levels were increased after crossing and at birth (**p < 0.001 vs. Control)

In DMO, body weight was reduced at P1 (**p < 0.01 vs. Control) and P7 (**p < 0.01 vs. Control) (Fig. 1a). Glucose levels (mg/dl) were slightly higher in DMO at P1 (**p < 0.01 vs. Control), and this effect recovered by day 7 (*p* = 0.171) (Fig. 1b). Slight reductions of insulin levels (ng/ml) in DMO did not reach statistical significance at P1 (*p* = 0.064) or P7 (*p* = 0.077) (Fig. 1c). By 5 weeks of age DMO body weight was still lower than the rest of the groups, and while ICV-Ins treatment significantly reduced this effect, only IN-Ins completely reversed the situation [F_(5,43)_ = 8.48, **p < 0.01 vs. rest of the groups] (Fig. 1a). By 10 weeks of age differences in body weight were no longer detectable [F_(5,79)_ = 1.16, *p* = 0.146] (Fig. 1a). Insulin levels were slightly lower in DMO by 5 and 10 weeks, although differences did not reach statistical significance and central insulin treatments did not have a significant effect on peripheral insulin levels (Fig. 1c). No differences were detected in glucose levels at 5 or 10 weeks after birth in DMO ([F_(5,46)_ = 0.579, *p* = 0.716] and [F_(5,79)_ = 0.955, *p* = 0.451] respectively) (Fig. 1b). However, GTT in 10 weeks old mice revealed altered glucose tolerance in DMO, while a complete recovery was observed after insulin treatments [F_(3,45)_ = 12.29, **p < 0.01 vs. rest of the groups] (Fig. 1d). Plasma insulin levels during GTT were significantly lower in DMO and insulin treatments corrected this effect [F_(5,47)_ = 10.32, **p < 0.01 vs. rest of the groups, ††*p* < 0.01 vs. Control + IN-Ins] (Fig. 1e).

Insulin levels in the brain were also tested after IN and ICV delivery. An overall increase of central insulin levels was observed in all regions under study. As it could be expected, higher insulin levels were observed in the olfactory bulb after in intranasal administration, while insulin levels were higher in the hippocampus and the striatum after ICV administration (Fig. 1f).

### Cognitive assessment

**Fig. 2:**
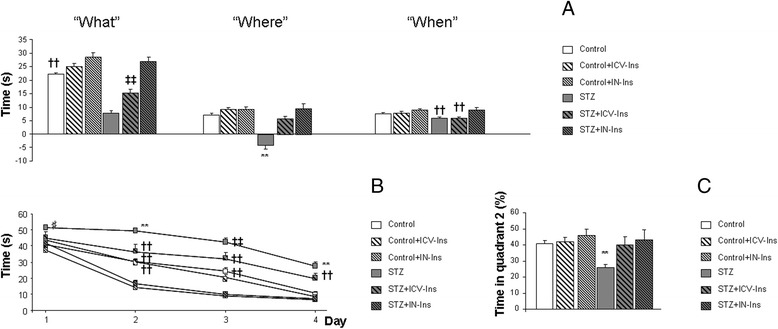
Learning and memory were significantly improved after insulin administration. **a** Episodic memory was affected in DMO and an overall improvement was observed after insulin administration in paradigms under study: “what” (**p < 0.01 vs. rest of the groups, ‡‡*p* < 0.01 vs Control, Control + IN-Ins, STZ + ICV-Ins, STZ + IN-Ins, ††*p* < 0.01 vs. Control + IN-Ins and STZ + IN-Ins), “where” (**p = 0.001 vs. rest of the groups), “when” (††*p* = 0.001 vs. Control + IN-Ins and STZ + IN-Ins) (Control *n* = 26, Control + ICV-Ins *n* = 19, Control + IN-Ins *n* = 8, STZ *n* = 15, STZ + ICV-Ins *n* = 12 and STZ + IN-Ins *n* = 6). **b** DMO were significantly impaied in the acquisition phase of the MWM and insulin treatment reversed this situation (day 1: ♯*p* = 0.012 vs. Control + IN-Ins, day 2: **p < 0.01 vs. rest of the groups, ††*p* < 0.01 vs. Control + IN-Ins and STZ + IN-Ins, day 3 ‡‡*p* < 0.01 vs. Control + IN-Ins, STZ-IN-Ins, Control and Control-ICV-Ins groups, ††*p* < 0.01 vs. Control + IN-Ins and STZ + IN-Ins, day 4: **p < 0.01 vs. rest of the groups, ††*p* < 0.01 vs. Control − +IN-Ins and STZ + IN-Ins). Two-way ANOVA was used to detect a groupXday interaction and one-way ANOVA, followed by Tuckey b or Tamhane test was used for further assessment of individual days. **c** Retention phase of the MWM also revealed an overall improvement after insulin administration to DMO (**p < 0.01 vs. rest of the groups) (Control *n* = 28, Control + ICV-Ins *n* = 17, Control + IN-Ins *n* = 8, STZ *n* = 17, STZ + ICV-Ins *n* = 10 and STZ + IN-Ins *n* = 6). Differences were detected by one-way ANOVA, followed by Tuckey b test

By 10 weeks, of age episodic memory was significantly impaired in DMO for all three paradigms under study: “what”, “where” and “when”. ICV-Ins recovered “what” and “where” limitations while IN-Ins completely reversed all three paradigms (“What” [F_(5, 252)_ = 48.55, **p < 0.01 vs. rest of the groups, ‡‡*p* < 0.01 vs Control, Control + IN-Ins, STZ + ICV-Ins, STZ + IN-Ins, ††*p* < 0.01 vs. Control + IN-Ins and STZ + IN-Ins]. “Where” [F_(5, 249)_ = 34.21, **p = 0.001 vs. rest of the groups]. “When” [F_(5, 245)_ = 4.49, ††*p* = 0.001 vs. Control + IN-Ins and STZ + IN-Ins] (Fig. 2a). A similar outcome was observed in the MWM test. The acquisition showed a significant treatmentXday effect [F_(15,1263)_ = 2.705, **p < 0.01]. Further assessment of individual days revealed an overall improvement in insulin treated mice. This effect was specially relevant in IN treated mice: day 1 [F_(5335)_ = 2.994, ♯*p* = 0.012 vs. Control + IN-Ins], day 2 [F_(5325)_ = 18.23, **p < 0.01 vs. rest of the groups, ††*p* < 0.01 vs. Control + IN-Ins and STZ + IN-Ins], day 3 [F_(5332)_ = 18.90, ‡‡*p* < 0.01 vs. Control + IN-Ins and STZ + IN-Ins, Control and Control + ICV-Ins groups, ††*p* < 0.01 vs. Control + IN-Ins and STZ + IN-Ins], day 4 [F_(5330)_ = 23.676, **p < 0.01 vs. rest of the groups, ††*p* < 0.01 vs. Control + IN-Ins and STZ + IN-Ins] (Fig. 2c). On the 24 h retention phase memory was significantly impaired, and improved after IN or ICV-Ins administration [F_(5,73)_ = 5.42, **p < 0.01 vs. rest of the groups] (Fig. 2d).

### Central atrophy, neuronal and synaptic alterations

**Fig. 3:**
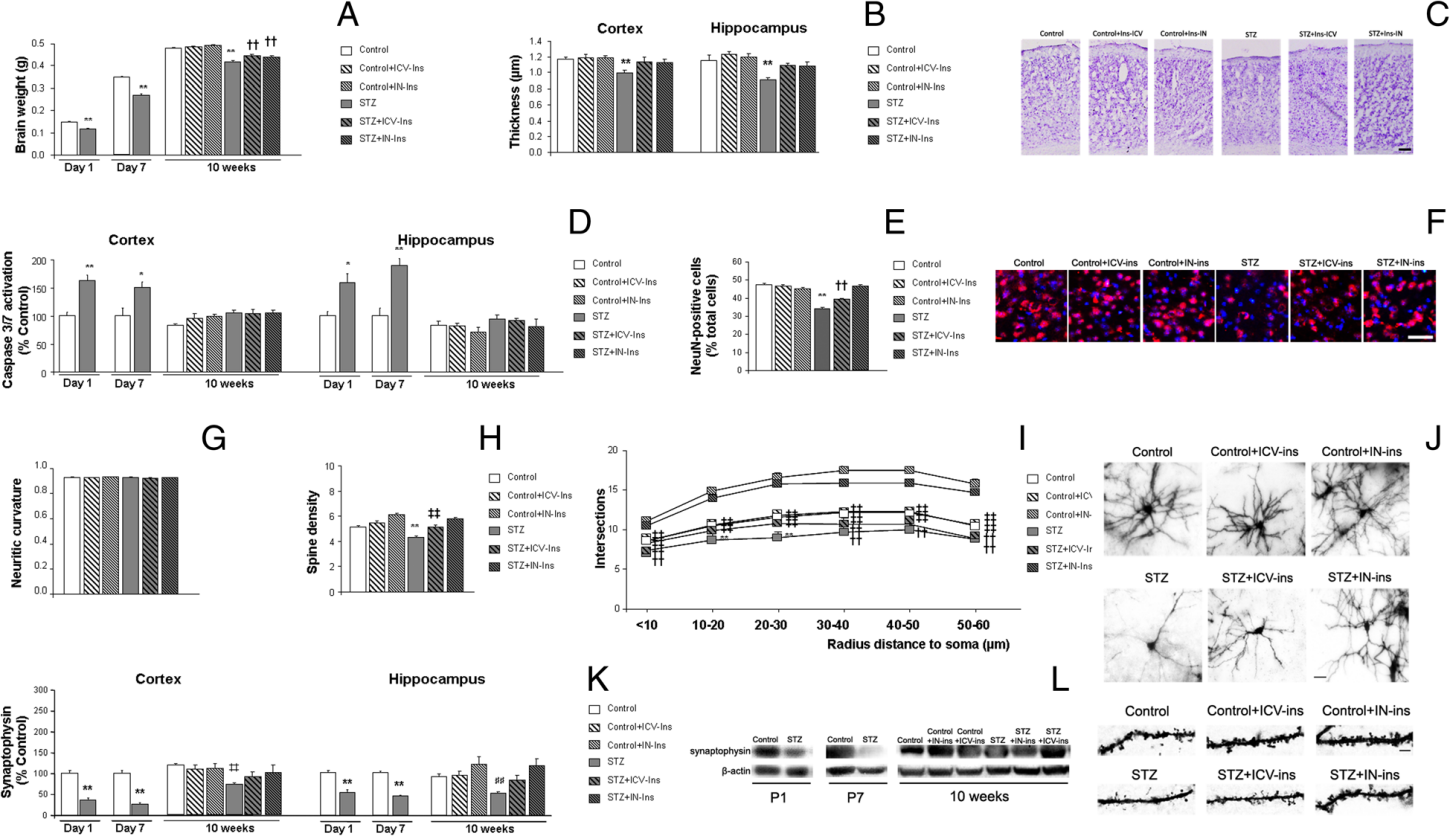
Insulin administration rescued central atrophy, neuronal and synaptic loss. **a** Brain weight was significantly reduced in DMO at P1 (**p < 0.01 vs. Control) and P7 (**p < 0.01 vs. Control). Insulin administration reversed this effect at 10 weeks of age (**p < 0.01 vs. rest of the groups, ††*p* < 0.01 vs. Control and Control + ICV-Ins) (P1 Control *n* = 6 and SZT *n* = 4; P7 Control *n* = 7 and STZ *n* = 9; 10 weeks Control *n* = 27, Control + ICV-Ins *n* = 17, Control + IN-Ins *n* = 8, STZ *n* = 18, STZ + ICV-Ins *n* = 10 and STZ + IN-Ins *n* = 6). **b** Cortical (**p = 0.03 vs. rest of the groups) and hippocampal (**p < 0.01 vs. rest of the groups) thickness are maintained after insulin treatment to DMO (*n* = 5 in all groups). **c** Illustrative example of cortical thickness after cresyl violet staining. Scale bar = 125 μm. **d** Cortical caspases 3/7 are activated in DMO at P1 (**p < 0.01 vs. Control) and P7 (*p = 0.016 vs. Control) while differences are no longer detectable at 10 weeks of age (*p* = 0.074). A similar profile is observed in the hippocampus at P1 (*p = 0.014 vs. Control), P7 (**p = 0.005 vs. Control) and at 10 weeks of age (*p* = 0.386) (P1a and P7 *n* = 9, 10 weeks *n* = 10 in all groups). **e** Insulin treatment maintained NeuN-positive cells in DMO (**p < 0.01 vs. rest of the groups, ††*p* < 0.01 vs. Control, Control + ICV-Ins, Control + IN-Ins and STZ+ IN-Ins) (Control *n* = 4, Control + ICV-Ins *n* = 5, Control + IN-Ins *n* = 5, STZ *n* = 5, STZ + ICV-Ins *n* = 5 and STZ + IN-Ins *n* = 5). **f** Illustrative example of NeuN immunostaining and DAPI counterstain. Scale bar = 125 μm. **g** Neuritic curvature was not affected in any of the groups under study (*p* = 0.649) (*n* = 4 in all groups). **h** Spine density was reduced in DMO and insulin treatment reverted this effect (**p < 0.01 vs. rest of the groups, ‡‡*p* < 0.01. vs. Control + IN-Ins and STZ + IN-Ins) (Control *n* = 4, Control + ICV-Ins *n* = 4, Control + IN-Ins *n* = 5, STZ *n* = 3, STZ + ICV-Ins *n* = 3 and STZ + IN-Ins *n* = 6). **i** Neuronal complexity was significantly reduced in DMO and insulin administration reversed this limitation (<10 μm: ††*p* < 0.01 vs. Control, Control + ICV-Ins, Control + IN-Ins, STZ + IN-Ins, ‡‡*p* < 0.01. vs. Control + IN-Ins and STZ + IN-Ins], 10–20 μm: **p < 0.01 vs. rest of the groups, ‡‡*p* < 0.01. vs. Control + IN-Ins and STZ + IN-Ins, 20–30 μm: **p < 0.01 vs. rest of the groups, ‡‡*p* < 0.01. vs. Control + IN-Ins and STZ + IN-Ins, 40–50 μm: ††*p* < 0.01 vs. Control, Control + ICV-Ins, Control + IN-Ins, STZ + IN-Ins, ‡‡*p* < 0.01. vs. Control + IN-Ins and STZ + IN-Ins, 50–60 μm: ††*p* < 0.01 vs. Control, Control + ICV-Ins, Control + IN-Ins, STZ + IN-Ins, ‡‡*p* < 0.01. vs. Control + IN-Ins and STZ + IN-Ins) Control *n* = 4, Control + ICV-Ins *n* = 4, Control + IN-Ins *n* = 5, STZ *n* = 3, STZ + ICV-Ins *n* = 3 and STZ + IN-Ins *n* = 6). **j** Illustrative example of neuronal complexity (Scale bar = 25 μm) and spine density (Scale bar = 10 μm). **l** Cortical synaptophysin levels were reduced in DMO at P1 (**p = 0.001 vs. control) and at P7 (**p < 0.001 vs. control). Insulin administration rescued synaptophysin levels at 10 weeks of age (‡‡*p* = 0.002 vs. Control, Control + ICV-Ins, Control + IN-Ins and STZ + IN-Ins) (P1 Control *n* = 6 and SZT *n* = 4; P7 Control *n* = 6 and STZ *n* = 6; 10 weeks Control *n* = 6, Control + ICV-Ins *n* = 6, Control + IN-Ins *n* = 5, STZ *n* = 6, STZ + ICV-Ins *n* = 6 and STZ + IN-Ins *n* = 4). A similar profile was observed in hippocamal synaptophysin levels at P1 (**p < 0.001 vs. control), P7 (**p < 0.001 vs. control) and 10 weeks of age (♯♯*p* = 0.06 vs. Control + IN-Ins and Control + IN-Ins) (P1, P7 and 10 weeks *n* = 6 in all groups). Student t-test for independent samples was used when 2 populations were under study (P1 and P7) and one-way ANOVA, followed by Tuckey b or Tamhane test, was used when 6 groups were under study (5 and 10 weeks). **k** Illustrative example of cortical synaptophysin levels in all groups under study

Brain weight was reduced in P1 DMO (**p < 0.01 vs. Control) and P7 (**p < 0.01 vs. Control) (Fig. 3a). At 10 weeks of age a partial recovery was observed in STZ + IN-Ins and STZ + ICV-Ins treated mice [F_(5,75)_ = 33.084, **p < 0.01 vs. rest of the groups, ††*p* < 0.01 vs. Control and Control + ICV-Ins] (Fig. 3a). Cortical thickness was reduced in DMO, while insulin treatments counterbalanced this effect [F_(5165)_ = 3.68, **p = 0.03 vs. rest of the groups] (Fig. 3b and c). A similar profile was observed in the hippocampus [F_(5,73)_ = 5.32 **p < 0.01 vs. rest of the groups] (Fig. 3b). Caspase activation assay, as a marker of cell death, was significantly increased in the cortex from DMO at P1 (**p < 0.01 vs. Control) and P7 (*p = 0.016 vs. Control) while differences were no longer observed at 10 weeks of age [F_(5,24)_ = 2.33, *p* = 0.074]. A similar profile was observed in the hippocampus at P1 (*p = 0.014 vs. Control) or P7 (**p = 0.005 vs. Control) (Fig. 3d).

NeuN-positive cells/total cells ratio was reduced in DMO, improved by ICV-Ins administration, and returned to control levels after IN-Ins treatment [F_(5,3435)_ = 75.81, **p < 0.01 vs. rest of the groups, ††*p* < 0.01 vs. Control, Control + ICV-Ins, Control + IN-Ins and STZ + IN-Ins] (Fig. 3e and f). While neurite curvature was not affected [F_(5,1062)_ = 0.649] (Fig. 3g) spines density was reduced in DMO, and it recovered after insulin administration ([F_(5473)_ = 17.32, **p < 0.01 vs. rest of the groups, ‡‡*p* < 0.01. vs. Control + IN-Ins and STZ + IN-Ins]) (Fig. 3f and j). Also, neurite simplification in DMO, recovered in insulin treated animals. Analysis of intersections by sholl analysis revealed a groupXradius distance effect [F_(25,3130)_ = 3.38, *p* < 0.01]. Further analysis in 10 μm steps revealed a reduction of dendrite branching in DMO that was increased after ICV-Ins and improved after IN-Ins treatment: <10 μm [F_(5532)_ = 39.69, ††*p* < 0.01 vs. Control, Control + ICV-Ins, Control + IN-Ins, STZ + IN-Ins, ‡‡*p* < 0.01. vs. Control + IN-Ins and STZ + IN-Ins], 10–20 μm [F_(5523)_ = 58.05, **p < 0.01 vs. rest of the groups, ‡‡*p* < 0.01. vs. Control + IN-Ins and STZ + IN-Ins], 20–30 μm [F_(5517)_ = 55.54**p < 0.01 vs. rest of the groups, ‡‡*p* < 0.01. vs. Control + IN-Ins and STZ + IN-Ins], 40–50 μm [F(5529) = 47.39, ††*p* < 0.01 vs. Control, Control + ICV-Ins, Control + IN-Ins, STZ + IN-Ins, ‡‡*p* < 0.01. vs. Control + IN-Ins and STZ + IN-Ins], 50–60 μm [F_(5529)_ = 47.3958, ††*p* < 0.01 vs. Control, Control + ICV-Ins, Control + IN-Ins, STZ + IN-Ins, ‡‡*p* < 0.01. vs. Control + IN-Ins and STZ + IN-Ins] (Fig. 3i and j). Synaptophysin was significantly reduced in the cortex from DMO at P1 (**p = 0.001 vs. Control) and P7 (**p = 0.001 vs. Control). At 10 weeks ICV insulin treatment improved this effect that reached statistical significance in IN treated animals [F_(5,27)_ = 4.97, ‡‡*p* = 0.002 vs. Control, Control + ICV-Ins, Control + IN-Ins and STZ+ IN-Ins] (Fig. 3k and l). A similar profile was observed in the hippocampus at P1 (**p < 0.001 vs. Control), P7 (**p < 0.001 vs. Control) and 10 weeks of age (F_(5,30)_ = 4.071, ♯♯*p* = 0.06 vs. Control + IN-Ins and STZ + IN-Ins) (Fig. 3k).

### Proliferation and neurogenesis

**Fig. 4:**
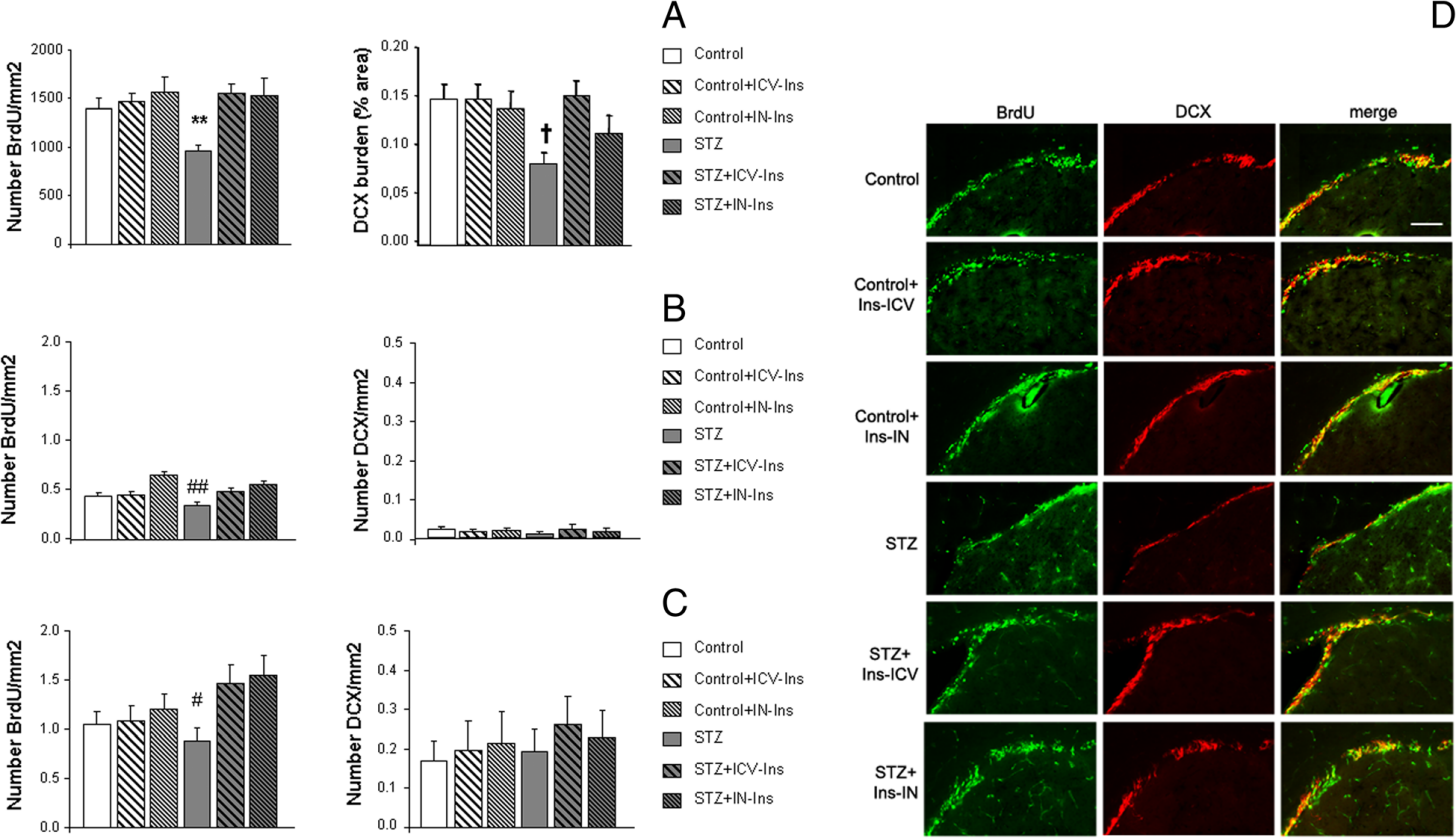
Insulin administration rescued proliferation and neurogenesis. **a** SVZ BrdU+ cells were reduced in DMO and insulin reversed this effect (**p = 0.07 vs. rest of the groups). DCX burden was also increased after insulin administration to DMO (†*p* = 0.019 vs. Control, Control + IN-Ins, Control + ICV-Ins and STZ + ICV-Ins) (Control *n* = 5, Control + ICV-Ins *n* = 5, Control + IN-Ins *n* = 4, STZ *n* = 5, STZ + ICV-Ins *n* = 3 and STZ + IN-Ins *n* = 3). **b** Cortical number of BrdU+ cells was reduced in DMO and insulin administration reversed this effect (##*p* < 0.01 vs. Control + IN-Ins and STZ + IN-Ins) while differences did not reach statistical significance when DCX+ cells were counted (*p* = 0.848). (Control *n* = 5, Control + ICV-Ins *n* = 5, Control + IN-Ins *n* = 4, STZ *n* = 5, STZ + ICV-Ins *n* = 3 and STZ + IN-Ins *n* = 3). **c** The same profile was observed when hippocampal BrdU+ (#*p* < 0.05 vs. STZ + IN-Ins) and DCX+ cells (*p* = 0.964) were analyzed (Control *n* = 5, Control + ICV-Ins *n* = 3, Control + IN-Ins *n* = 4, STZ *n* = 5, STZ + ICV-Ins *n* = 3 and STZ + IN-Ins *n* = 3). One-way ANOVA followed by Tuckey b or Tamhane tests, was used for statistical analysis. **d** Representative images from the SVZ where reduced BrdU+ (*green*) and DCX+ (*red*) staining can be observed in DMO, while ICV-Ins and IN-Ins reversed the situation

In the SVZ the number of BrdU+ cells was significantly compromised in DMO (Fig. 4a and d) and a complete recovery was observed after insulin treatment [F_(5,78)_ = 3.5, **p = 0.07 vs. rest of the groups]. DCX burden was also reduced DMO and ICV insulin treatment reversed this situation [F_(5,74)_ = 2.91, †*p* = 0.019 vs. Control, Control + IN-Ins, Control + ICV-Ins and STZ + ICV-Ins] (Fig. 4a and d). BrdU+ cells were significantly reduced in the cortex from DMO. This situation improved by ICV-Ins administration and completely recovered after IN-Ins [F_(5208)_ = 7.87, ##*p* < 0.01 vs. Control + IN-Ins and STZ + IN-Ins]. A similar profile was observed when we quantified DCX-positive cells, although differences did not reach statistical significance ([F_(5208)_ = 0.4, *p* = 0.848]) (Fig. 4b). A similar profile was observed in the hippocampus for BrdU-positive cells [F_(5118)_ = 2.59 #*p* < 0.05 vs. STZ + IN-Ins] and. DCX-positive cells ([F_(5122)_ = 0.195, *p* = 0.964]) (Fig. 4c).

### Akt levels

**Fig. 5:**
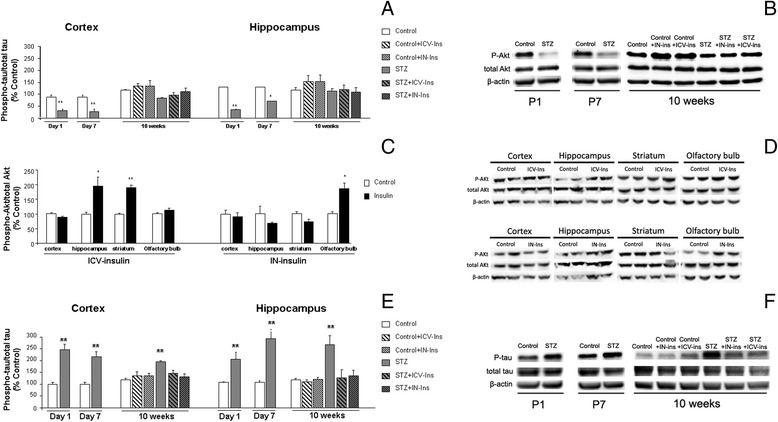
Effect of insulin administration on P-Ak/total Akt and P-tau/total tau and ratios. **a** Cortical Akt phosphorylation was reduced in DMO at P1 (**p = 0.001 vs. control) and P7 (**p < 0.001 vs. control). At 10 weeks of age insulin administration slightly increased Akt phosphorylation although differences are not statistically significant (*p* = 0.066) (P1 Control *n* = 6 and SZT *n* = 4; P7 Control *n* = 6 and STZ *n* = 6; 10 weeks Control *n* = 6, Control + ICV-Ins *n* = 5, Control + IN-Ins *n* = 6, STZ *n* = 6, STZ + ICV-Ins *n* = 6 and STZ + IN-Ins *n* = 6). A similar profile was observed in the hippocampus at P1 (*p < 0.01 vs. control), P7 (*p = 0.035 vs. control) and 10 weeks (*p* = 0.390) (P1 *n* = 6 in all groups, P7 *n* = 5 in all groups, 10 weeks *n* = 6 in all groups). **b** Illustrative example of phospho-Akt, total Akt and β-actin westerns blots. **c** Phospho-Akt/total AKt ratios were significantly increased 4 h after insulin administration in regions located in the proximity of the administration site: the hippocampus and the striatum after ICV administration (cortex *p* = 0.092, hippocampus *p = 0.045 vs. Control, striatum **p < 0.01 vs. Control, olfactory bulb *p* = 0.148), and the olfactory bulb after IN administration (cortex *p* = 0.641, hippocampus *p* = 0.353, striatum p0.063, olfactory bulb *p* = 0.022). **d** Illustrative example of phospho-Akt, total Akt and β-actin westerns blots after IN and ICV insulin administration. **e** Cortical tau phosphorylation was increased in DMO at P1(**p = 0.003 vs. Control) and P7 (**p = 0.001 vs. Control). Insulin administration significantly reduced tau hyperphosporylation at 10 weeks of age (**p < 0.01 vs. rest of the groups) (P1 Control *n* = 6 and SZT *n* = 4; P7 Control *n* = 6 and STZ *n* = 6; 10 weeks Control *n* = 6, Control + ICV-Ins *n* = 6, Control + IN-Ins *n* = 6, STZ *n* = 6, STZ + ICV-Ins *n* = 5 and STZ + IN-Ins *n* = 6). A similar profile was observed in the hippocampus at P1 (**p = 0.007 vs. control), P7 (**p = 0.001 vs. control) and 10 weeks of age (**p < 0.01 vs. rest of the groups) (P1 Control *n* = 6 and SZT *n* = 4; P7 Control *n* = 6 and STZ *n* = 6; 10 weeks Control *n* = 6, Control + ICV-Ins *n* = 6, Control + IN-Ins *n* = 6, STZ *n* = 6, STZ + ICV-Ins *n* = 5 and STZ + IN-Ins *n* = 6)**.** One-way ANOVA, followed by Tuckey b or Tamhane test, was used for statistical analysis. **f** Illustrative example of phospho-tau, total tau and β-actin westerns blots

Phopho-Akt/total Akt levels were reduced in the cortex of DMO at P1 (**p = 0.001 vs. Control) and P7 (**p < 0.001 vs. Control). By 10 weeks of age, phospho-Akt levels were still reduced in DMO and insulin administration slightly increased the ratio, although differences were not statistically significant [F_(5,29)_ = 2.34, *p* = 0.066] (Fig. 5a and b). A similar profile was observed in the hippocampus at P1 (**p < 0.01 vs. Control), P7 (**p < 0.01 vs. Control) and by 10 weeks of age [F_(5,30)_ = 1.98, *p* = 0.390] (Fig. 5a). Since it is feasible that the time selected after insulin delivery might be too long we also measured Phopho-Akt/total Akt ratio 4h after ICV or intranasal administration. Phospho-Akt/total Akt ratios were significantly increased in those regions located in the proximity of the administration site: the hippocampus and the striatum after ICV administration (cortex *p* = 0.092, hippocampus *p = 0.045 vs. Control, striatum **p < 0.01 vs. Control, olfactory bulb *p* = 0.148), and the olfactory bulb after intranasal administration (cortex *p* = 0.641, hippocampus *p* = 0.353, striatum *p* = 0.063, olfactory bulb *p = 0.022) (Fig. 5c and d).

### Tau pathology

Tau phosphorylation was significantly increased in DMO at P1 (**p = 0.03 vs. Control) and P7 (**p < 0.01 vs. Control). This effect was still detectable after 10 weeks, and it was completely reversed by insulin administration [F_(5,29)_ = 5.25, **p < 0.01 vs. rest of the groups] (Fig. 5e and f). A similar profile was observed in the hippocampus (P1, **p = 0.007 vs. Control; P7, **p < 0.001 vs. Control and 10 weeks of age [F_(5,29)_ = 6.68, **p < 0.01 vs. rest of the groups] (Fig. 5e).

### Spontaneous bleeding

**Fig. 6:**
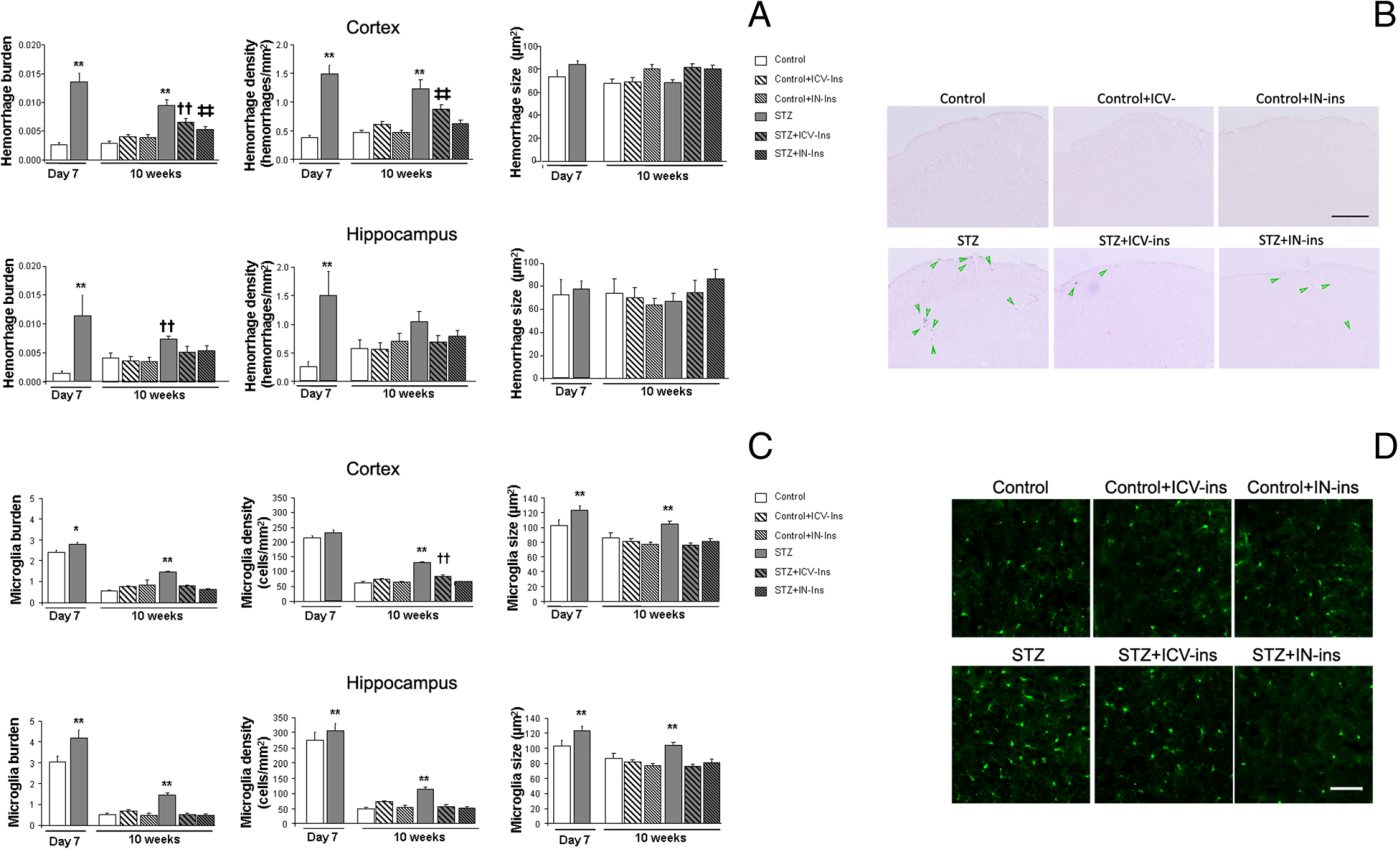
Effect of insulin administration on central spontaneous bleeding and inflammation. **a** Cortical haemorrhage burden was significantly increased in DMO at P7 (**p < 0.01 vs. Control). By 10 weeks of age insulin administration significantly ameliorated this effect (**p < 0.01 vs. 0.01 vs. rest of the groups, ††*p* < 0.01 vs. Control, Control + ICV-Ins, Control + IN-Ins, ‡‡*p* < 0.01 vs. Control). A similar profile was observed when we measured hemorrahge density (P7: **p < 0.01 vs. Control; 10 weeks: **p < 0.01 vs. 0.01 vs. rest of the groups, ‡‡*p* < 0.01 vs. Control) while no differences were observed in hemorrhage size (P7: *p* < 0.053 vs. Control; 10 weeks *p* = 0.01 and no further differences were detected by Tuckey b test) (P7 *n* = 5 both groups, 10 weeks Control *n* = 5, Control + ICV-Ins *n* = 5, Control + IN-Ins *n* = 5, STZ *n* = 4, STZ + ICV-Ins *n* = 6 and STZ + IN-Ins *n* = 5). Hippocampal haemorrhage burden was increased in DMO and insulin administration reduced this effect by 10 weeks of age (P7: **p < 0.01 vs. Control; 10 weeks: ††*p* < 0.01 vs. Control, Control + ICV-Ins, Control + IN-Ins). A similar profile was observed in hemorrhage density although differences did not reach statistical significance at 10 weeks of age (P7: **p = 0.05 vs. Control, 10 weeks *p* = 0.165). No differences were observed in hemorrhage size (P7: *p* = 0.184; 10 weeks *p* = 0.410) (P7 *n* = 5 both groups, 10 weeks Control *n* = 5, Control + ICV-Ins *n* = 5, Control + IN-Ins *n* = 5, STZ *n* = 4, STZ + ICV-Ins *n* = 6 and STZ + IN-Ins *n* = 5). **b** Illustrative example of cortical haemorrhages at 10 weeks of age stained with Prussian blue. Scale bar = 500 μm. **c** Cortical microglia burden was increased at P7 (*p = 0.018 vs. Control) and insulin administration limited this effect in DMO at 10 weeks of age (**p < 0.01 vs. rest of the groups), microglia size (P7: **p < 0.01 vs. Control; 10 weeks **p < 0.01 vs. rest of the groups). A similar profile was observed in microglia density (P7: *p* = 0.166; 10 weeks **p < 0.01 vs. rest of the groups, ††*p* < 0.01 vs. Control, Control + ICV-Ins, Control + IN-Ins and STZ + IN-Ins) and microglia size (P7: **p < 0.01 vs. rest of the groups; 10 weeks **p < 0.01 vs. rest of the groups) (P7 Control *n* = 6 and STZ *n* = 5; 10 weeks Control *n* = 5, Control + ICV-Ins *n* = 5, Control + IN-Ins *n* = 4, STZ *n* = 5, STZ + ICV-Ins *n* = 3 and STZ + IN-Ins *n* = 3). A similar profile was observed for hippocampal microglia burden (P7: **p < 0.01 vs. Control; 10 weeks: **p < 0.01 vs. rest of the groups), density (P7: **p = 0.005 vs. Control; 10 weeks **p < 0.01 vs. rest of the groups) and size (P7: **p < 0.01 vs. Control; 10 weeks **p < 0.01 vs. rest of the groups) (P7 Control *n* = 4 and STZ *n* = 5, 10 weeks *n* = 5 in all groups). One-way ANOVA, followed by Tuckey b or Tamhane test, was used for statistical analysis. **d** Illustrative example of cortical microglia immunostaining with iba-1 antibody. Scale bar = 50 μm

Cortical haemorrhage burden in DMO at P7 (**p < 0.01 vs. Control) and density (**p < 0.01 vs. Control) were increased, without affecting individual haemorrhage size (*p* = 0.053) (Fig. 6a). By 10 weeks of age haemorrhage burden was significantly higher in DMO and insulin administration significantly reduced this effect [F_(5153)_ = 16.052, **p < 0.01 vs. 0.01 vs. rest of the groups, ††*p* < 0.01 vs. Control, Control + ICV-Ins, Control + IN-Ins, ‡‡*p* < 0.01 vs. Control], by reducing haemorrhage density (number haemorrhages/mm^2^) [F_(5163)_ = 15.54, **p < 0.01 vs. 0.01 vs. rest of the groups, ‡‡*p* < 0.01 vs. Control] whereas haemorrhage size was not affected [F_(5943)_ = 4.12, *p* = 0.01 and no further differences were detected by Tuckey b test] (Fig. 6a and b). A similar profile was observed in the hippocampus when we analyzed haemorrhage burden (**p = 0.005 vs. Control), haemorrhage density (**p = 0.005 vs. Control) and haemorrhage size (*p* = 0.184) at P7 (Fig. 6a) or at 10 weeks of age, (burden:[F_(5,61)_ = 16.052, ††*p* < 0.01 vs. Control, Control + ICV-Ins, Control + IN-Ins], density: [F_(5,68)_ = 1.62, *p* = 0.165] and size [F_(5119)_ = 1.01, *p* = 0.410]) (Fig. 6a).

### Inflammation

At P7 microglia burden (*p = 0.018 vs. Control) and microglia size (**p < 0.01 vs. Control) were increased in the cortex. Microglia density was not affected (*p* = 0.166) (Fig. 6c). By 10 weeks of age, increased inflammation in DMO was completely reversed by insulin administration [F_(5,3437)_ = 155.16, **p < 0.01 vs. rest of the groups, ††*p* < 0.01 vs. Control, Control + IN-Ins and STZ + IN-Ins]. Microglia density [F(_5,3429)_ = 92.12, **p < 0.01 vs. rest of the groups, ††*p* < 0.01 vs. Control, Control + IN-Ins, Control + ICV-Ins and STZ + IN-Ins] and microglia size [F(_5,3665)_ = 28.91, **p < 0.01 vs. rest of the groups] were also reduced (Fig. 6c and d). A similar profile was observed in the hippocampus at P7 (microglia burden: **p < 0.01 vs. Control, size **p < 0.01 vs. Control and number: **p < 0.01 vs. Control) (Fig. 6c) and at 10 weeks of age (burden: [F_(5537)_ = 31.79, **p < 0.01 vs. rest of the groups], density: [F_(5535)_ = 16.10, **p < 0.01 vs. rest of the groups] and size: [F_(5480)_ = 8.67, **p < 0.01 vs. rest of the groups]) (Fig. 6c).

## Discussion

Increased risk of metabolic and cardiovascular diseases in DMO have been reported in animal and human [24] studies. While cognitive alterations in DMO have also been observed [9, 10], it is noteworthy that most of previous studies are based on observational cohorts. Therefore a direct causal influence of intrauterine hyperglycemia remains uncertain [25] and therapeutic options have not been completely addressed. To help elucidate some of these aspects, we have analyzed the short and long-term central complications in a murine model of DMO, as well as the effects of ICV and non-invasive IN insulin treatments. Our approach included administering STZ to mothers before crossing [26], and central complications in DMO have been also observed when mothers are mated and diabetes is induced immediately afterwards [27]. However we cannot exclude that final outcomes might be slightly different depending on the protocol used, time selected to induce diabetes or endpoints under study.

The fact that glucose levels recovered 7 days after birth might be due to regularization of basal levels once the pups are no longer in intrauterine environment. Increased insulin levels did not reach statistical significance, however it remains feasible that the slight a hyperglycemic increase observed might be enough to control glycaemia in our population. In DMO we also observed growth inhibition, impaired GTT and insulin response, as previously reported in DMO, that show decreased insulin sensitivity and low insulin secretion [28]. The fact that DMO do not regularly release insulin in the GTT suggests a pancreatic exhaustion, over insulin resistance. Nevertheless, we cannot unequivocally point towards a single cause leading to observed metabolic alterations. On the other hand, central insulin administration reversed all these effects, supporting the role of insulin in the regulation of peripheral metabolic complications [29, 30].

Central pathology in young adults from diabetic mothers revealed the long term effects of severe maternal hyperglycemia in learning and memory. Epidemiological studies have shown that maternal diabetes is negatively associated with offspring’s cognitive development [10] and gestational diabetes has been related to lower general intelligence, language impairment, attention weakness, impulsivity or behavioural problems (for review [9]). A few studies have assessed the ong-term effects of maternal diabetes in the descendants [31] and to our knowledge this is the first one trying to reverse observed deficits by administering insulin to the offspring. Spatial and episodic memory impairment in DMO improved after ICV insulin administration, and were completely reversed after IN insulin. While ICV administration provides useful tool to guarantee access to the CNS in animal models [30], its translational approach is not feasible. On the other hand, IN insulin uses a transport system, via the nasal epithelium into the brain, that avoids high levels of insulin in the periphery. Moreover, previous studies with IN insulin support that insulin signal is crucial in neuronal function and cognition, improving cognitive function in healthy subjects [32], diabetic patients [33] and murine models [34], however to our knowledge no previous studies have tested insulin administration to restore cognitive deficits associated to intrauterine hyperglycaemia.

Significant brain atrophy was observed in DMO up to early adulthood, associated with increased caspase activity, cortical and hippocampal thinning, in line with previous studies [35]. Likewise, NeuN/DAPI ratios were reduced in DMO, while insulin treatment completely reversed these observations. In order to further characterize brain atrophy, we examined neuronal morphology, since it has been shown to correlate with neuronal function [36]. Neurite curvature was not significantly altered in DMO, however, severe reduction of neuronal ramifications, spine density and synaptophysin levels were observed, in accordance with previous studies [37]. Synaptogenesis is a relevant event during development that requires a fine and precise pre- and postsynaptic specialization [35]. Therefore, interferences at this level may result in abnormal layer development and related cognition and behavioural problems. Insulin treatment fully recovered neuronal complexity and spine density in DMO, in line with previous studies showing that altered maternal metabolism may impair offspring neuronal projections normal development [38]. It has been already reported that foetal hyperglycaemia alters the expression of genes involved in proliferation and differentiation of neural cells [35]. Insulin also plays a crucial role in proliferation and neurogenesis in the CNS and in our hands, DMO presented a significant reduction of both processes, especially relevant in a neurogenic niche as the SVZ. Insulin signaling in the brain can respond to changes in systemic metabolic state while local insulin signaling promotes neurogenesis [39], as observed in our DMO after insulin treatment. Since Akt plays a crucial role in insulin signaling, as well as in cell proliferation and cell survival, it has been previously suggested that variations in Akt may underlie the observed alterations in DMO [40]. We also observed a reduction in phospho-Akt levels in young DMO, supporting that diabetes during pregnancy strongly influences the regulation of Akt in the developing brain, and that strict maternal metabolic control might be crucial [26]. Insulin treatment slightly increased phospho-Akt levels. Limited differences observed might be due to the fact that determinations were performed days and weeks after insulin administration. Therefore, we also performed determinations 4 h after insulin administration and we detected that phospho-Akt/total AKt ratios were significantly increased in those regions located in the proximity of the administration site: the hippocampus and the striatum after ICV administration, and the olfactory bulb after intranasal administration, in line with previous observations [41].

Tau hyperphosphorylation exerts toxic effects and it is a pathological hallmark of neurodegenerative disorders, also described in different diabetic animal models [17, 19]. It has been suggested that hyperglycaemic conditions induce tau deregulation [42]. We observed a significant increase of hyperphophorylated tau in DMO and similar outcomes have been observed in other models [43]. However, to our knowledge, the detected improvement after insulin treatment has not been previously reported.

Small vessel disease is a more common cause of ischemic stroke in people with diabetes [44]. It has been also described that embryos from diabetic mothers present generalized vascular lesions [45], however we believe no previous studies have assessed spontaneous central bleeding in DMO. Haemorrhage burden was increased in P7 DMO, and this effect was still detectable in the adulthood, supporting the long-term effects of maternal diabetes in the offspring. Previous studies have also associated the presence of haemorrhages with an increase in tau phosphorylation [46, 47]. Moreover, insulin treatment can effectively reverse these effects. We also detected a significant increase in microglia burden up to early adulthood in DMO, in line with previous studies showing that maternal prediabetes is enough to increase microglia activation and cytokines involved with trafficking across the blood–brain barrier [48], suggesting an activation of the local inflammatory response that may ultimately contribute to observed spontaneous bleeding. Furthermore, insulin treatment reduced microglia activation, supporting its role as an anti-inflammatory agent (for review see [49]).

## Conclusions

These data suggest that maternal diabetes not only affects the CNS development but it also has long-term effects, that are still evident in the adulthood. Maternal diabetes affects neuronal complexity and synaptic density, tau hyperphosphorylation, central inflammation or spontaneous bleeding, which may altogether compromise learning and memory abilities. Moreover, ICV insulin administration counterbalances many of these aspects and non-invasive IN administration robustly reverses detected alterations. This could lead to studies in greater depth on the use of IN insulin as a therapeutic alternative for those infants from diabetic mothers, who may benefit from more exhaustive follow-up assessments [9] and individualized treatments.

## Abbreviations

BrdU: 5-bromo-3-deoxyuridine
CNS: Central nervous system
DCX: doublecortin
DMO: diabetic mothers offspring
GTT: glucose tolerance test
ICV: intracerebroventricular
ICV-Ins: Intracerebroventricular insulin
IN: intranasal
IN-Ins: Intranasal insulin
MWM: Morris water maze
NOD: new object discrimination
STZ: streptozotozin
SVZ: subventricular zone
